# SINEs as Potential Expression Cassettes: Impact of Deletions and Insertions on Polyadenylation and Lifetime of B2 and Ves SINE Transcripts Generated by RNA Polymerase III

**DOI:** 10.3390/ijms241914600

**Published:** 2023-09-27

**Authors:** Olga R. Borodulina, Ilia G. Ustyantsev, Dmitri A. Kramerov

**Affiliations:** Laboratory of Eukaryotic Genome Evolution, Engelhardt Institute of Molecular Biology, Russian Academy of Sciences, 32 Vavilov St., Moscow 119991, Russia; olgarb@mail.ru (O.R.B.); usian@mail.ru (I.G.U.)

**Keywords:** SINE, retroposon, retrotransposon, RNA polymerase III, polyadenylation, RNA stability, non-coding RNA, expression cassette, mammals, cell culture

## Abstract

Short Interspersed Elements (SINEs) are common in the genomes of most multicellular organisms. They are transcribed by RNA polymerase III from an internal promoter comprising boxes A and B. As transcripts of certain SINEs from mammalian genomes can be polyadenylated, such transcripts should contain the AATAAA sequence as well as those called β- and τ-signals. One of the goals of this work was to evaluate how autonomous and independent other SINE parts are β- and τ-signals. Extended regions outside of β- and τ-signals were deleted from SINEs B2 and Ves and the derived constructs were used to transfect HeLa cells in order to evaluate the relative levels of their transcripts as well as their polyadenylation efficiency. If the deleted regions affected boxes A and B, the 5′-flanking region of the U6 RNA gene with the external promoter was inserted upstream. Such substitution of the internal promoter in B2 completely restored its transcription. Almost all tested deletions/substitutions did not reduce the polyadenylation capacity of the transcripts, indicating a weak dependence of the function of β- and τ-signals on the neighboring sequences. A similar analysis of B2 and Ves constructs containing a 55-bp foreign sequence inserted between β- and τ-signals showed an equal polyadenylation efficiency of their transcripts compared to those of constructs without the insertion. The acquired poly(A)-tails significantly increased the lifetime and thus the cellular level of such transcripts. The data obtained highlight the potential of B2 and Ves SINEs as cassettes for the expression of relatively short sequences for various applications.

## 1. Introduction

SINEs or short retrotransposons are non-autonomous mobile genetic retroelements shorter than 600 bp that are transcribed by RNA polymerase III (pol III) (reviewed in [1,2,3,4]). SINEs are typical for the majority of multicellular organisms and the number of their genomic copies can be as high as a million [3,4]. SINE families are amplified by reverse transcription (retrotransposition) mediated by an enzyme encoded in Long Interspersed Elements (LINEs). Most SINE families originated from tRNAs so that their 5′-terminal (head) regions typically have high sequence similarity to a particular tRNA species. Both tRNA genes and their SINE derivatives are transcribed by pol III from the corresponding internal promoter, which consists of 11-nt boxes A and B separated by 30–40 bp. The 3′-terminal region of SINEs typically consists of the sequence recognized by the LINE reverse transcriptase. Most SINE families in placental mammals rely on the reverse transcriptase of LINE1 (L1) and require a 3′-terminal poly(A) tail for retrotransposition.

SINEs are important factors in genome evolution and function. Their integration into genes including their introns and regulatory regions can affect gene function, i.e., induce deleterious mutations [3,5,6]. At the same time, many other SINE insertions into introns or regulatory regions do not induce negative effects on gene function but can modulate it and upregulate transcription [7,8,9] as well as splicing [3,10,11] or polyadenylation [12,13] of mRNA. Furthermore, SINE transcripts synthesized by pol III can potentially counteract cell stress [14,15].

While studying SINE families from mammalian genomes, we noticed that some of them had the polyadenylation signal AATAAA (PAS) and pol III transcription terminators (TCT ≥ 3 or T ≥ 4) upstream of the poly(A) tail [16]. These T-rich signals led us to assign such SINEs to the T^+^ class, while SINEs without these signals were considered as T^─^ SINEs. All 12 T^+^ SINE families are found exclusively in placentals [4,16,17]. Previously, such polyadenylation was considered specific for RNA polymerase II transcripts, primarily mRNAs. Experiments with B2, Dip, and Ves SINEs (from the mouse, jerboa, and bat genomes, respectively) revealed two additional motifs contributing to polyadenylation besides PAS: β-signal is located immediately downstream of box B of the pol III promoter, while τ-signal precedes the PAS region [18]. In B2 transcripts, the τ-signal is the binding site for the polyadenylation factor CFIm that facilitates the association of a protein subcomplex mPSF with AAUAAA [17]. In Dip and Ves, polypyrimidine motifs function as τ-signals [18]; similar polypyrimidine motifs are typical of four other T^+^ SINE families. The mechanism of the contribution of polypyrimidine motifs in polyadenylation of Dip and Ves transcripts remains unknown. The same applies to the β-signal in the B2 transcript. A long poly(A) tail (A > 20) in SINE transcripts is strictly required for the retrotransposition mediated by L1 reverse transcriptase [19,20,21]. In addition, 3′-terminal poly(A) tails in pol III transcripts of T^+^ SINEs substantially prolong the lifetime of such transcripts in the cell [22,23].

The goal of this work was to evaluate the extent to which the β- and τ-signals in B2 and Ves can function autonomously and independently of other SINE parts. In this context, it was of interest to determine whether transcripts of these SINEs could be polyadenylated without the internal promoter (boxes A and B) but with an external pol III promoter. This promoter of the U6 small nuclear RNA is composed of the proximal and distal sequence elements as well as the TATA-box (Figure S1) and initiates transcription of the downstream sequence [24,25,26]. Another goal was to test whether these SINEs with extended nucleotide sequence substitutions could be efficiently transcribed and the transcript could be polyadenylated in transfected cells. The positive results could substantiate the development of transcription cassettes for relatively short non-coding RNAs based on T^+^ SINEs. Such cassettes can be used to generate transcripts containing specific protein-binding sites, ribozymes, microRNA targets and competitors, hairpin precursors of siRNAs, etc. So far, tRNA, 5S RNA, 7SL RNA, and VA RNA genes as well as promoters of U6 RNA and H1 RNA have been used in this context [27,28,29,30,31,32]. All of these genes as well as SINEs are transcribed by pol III; however, the T^+^ SINE-based constructs can be polyadenylated, which increases the level and stability of such RNAs in the cell.

## 2. Results

### 2.1. Effect of U6 Promoter as Well as Deletions and Substitutions in B2 on the Level of Transcripts and Their Polyadenylation

**Figure 1:**
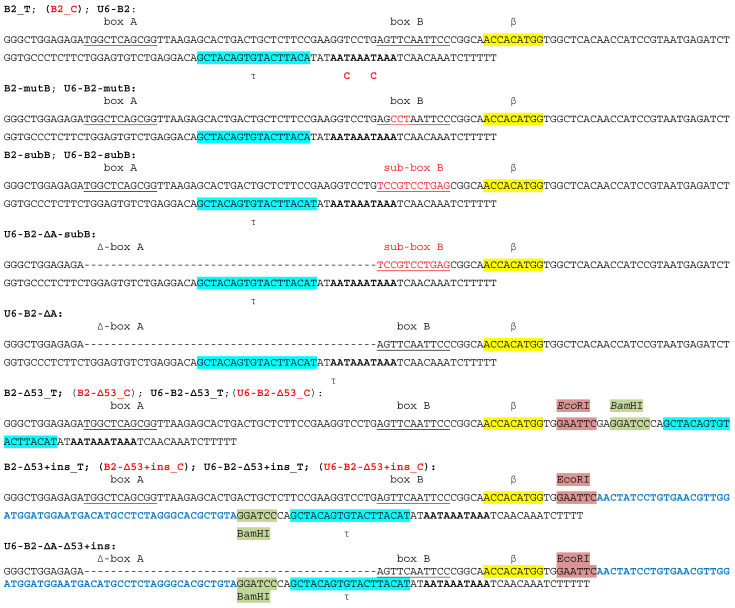
Nucleotide sequences of B2-containing constructs. Boxes A and B of the pol III promoter are underlined. The β- and τ-signals are marked in yellow and blue, respectively. The replaced nucleotides are marked in red; the deleted ones are indicated by dashes. In B2-Δ53A, the 53-bp fragment was substituted with *Eco*RI (brown) and *Bam*HI (green) sites. In B2-Δ53+ins, a random 55-bp sequence shown in blue was inserted between these sites. In the constructs with names beginning with ‘U6,’ the murine 5′-flanking sequence was replaced with the pol III promoter of the human U6 RNA gene, as shown by the corresponding nucleotide sequences in Figure S1. The PASs are indicated in bold. The constructs where PASs were inactivated by T to C substitution end with ‘C’ and are displayed in red.

A series of B2 constructs were generated (Figure 1) based on two constructs: B2_T, one of the B2 genomic copies with the 86-bp 5′-flanking sequence, and B2_C with both PASs inactivated by the T to C substitutions [18,22,33]. In the constructs whose names begin with “U6”, the mouse 5′-flanking sequence was replaced by the 261-bp 5′-flanking sequence of the human U6 RNA gene with the external type 3 promoter of pol III (Figure 1 and Figure S1). The names containing “mutB” or “subB” correspond to constructs with a TCC > CCT mutation or a substitution of all 11 bp in box B. “ΔA” indicates the deletion of box A and the 33-bp spacer between boxes A and B (Figure 1). Finally, “Δ53” indicates the replacement of the 53-bp region between the β- and τ-signals with a 14-bp sequence containing *Eco*RI and *Bam*HI sites.

The purpose of obtaining the constructs presented in Figure 1 was as follows. We wanted to investigate whether extended deletions or substitutions in SINE B2, including those affecting boxes A and B of the pol III promoter, change the efficiency of expression and polyadenylation of transcripts. The results of testing these constructs should shed light on: (1) to what extent β- and τ-signals are able to function after an extensive change in the nucleotide sequence of SINE B2 and (2) which regions in B2 could be replaced with foreign ones so that this SINE could be used as an expression cassette.

**Figure 2:**
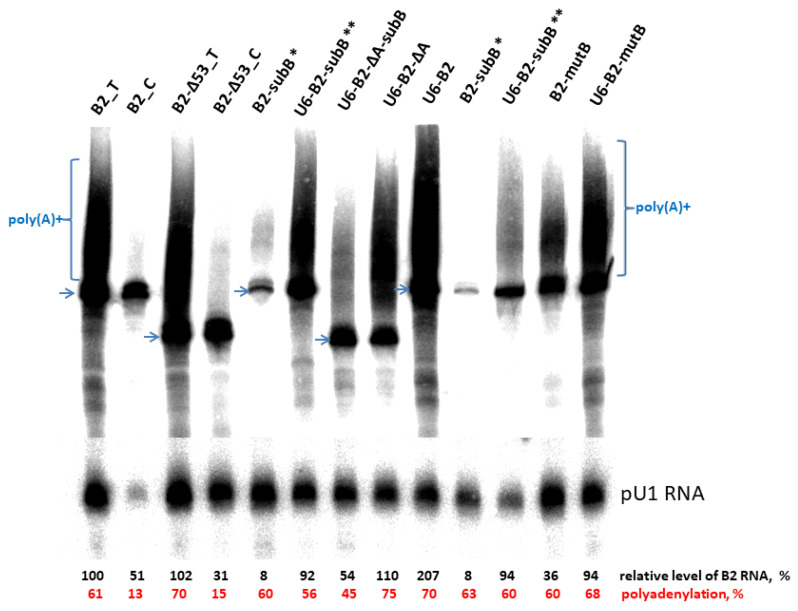
Northern hybridization of RNA from HeLa cells transfected by B2 constructs. The positions of the primary transcripts are marked by blue arrows, while polyadenylated transcripts are marked using blue brackets. Normalization was carried out using pU1 RNA. The level of B2 RNA relative to the B2_T RNA level is provided below as a percentage. The polyadenylation efficiency is shown in red at the bottom. The asterisks indicate transfections that were performed twice with independently isolated plasmid samples.

The resulting constructs were used to transfect HeLa cells together with the control plasmid pU1 [34], which is transcribed by pol II yielding 100-nt RNA. Cellular RNA was isolated and analyzed by PAGE followed by Northern hybridization (Figure 2). The total level of B2 transcripts and the proportion of polyadenylated B2 transcripts were evaluated (indicated at the bottom of Figure 2). Replacement of the region between the β- and τ-signals (B2-Δ53_T) did not decrease the level of B2 RNA relative to the original B2_T construct (taken as 100%), while the level of polyadenylation of B2-Δ53_T increased slightly (70 versus 61%). The cellular level of the B2-Δ53_C transcript with inactivated PASs was three times lower compared to the polyadenylation-competent B2-Δ53_T transcript. This confirms our idea that poly(A) tails slow down RNA degradation and increase its cellular content.

A trinucleotide mutation in box B (B2-mutB) decreased the level of B2 RNA 2.8-fold, whereas an external pol III promoter introduced into this construct (U6-B2-mutB) almost completely restored the transcript level (Figure 2). Replacement of all nucleotides in box B (B2-subB) decreased the RNA level 12.5-fold, and the U6 promoter introduced into the construct (B2_T) restored the B2 transcript level to 92–94% of baseline. Notably, the entire box B substitution had only a minor effect on the polyadenylation capacity of B2 RNA. The U6-B2 construct has two functional pol III promoters (external and internal), which doubled the B2 RNA level. Deletion of box A and the 33-bp spacer between boxes A and B in the U6-B2-ΔA construct results in a transcription level similar to that of the original B2_T, and the transcript polyadenylation is even more efficient compared to B2 RNA (75% versus 61%, respectively). Subsequent substitution of the entire box B (U6-B2-ΔA-subB) reduced the transcript level two-fold; and the polyadenylation efficiency, by 45%. Conceivably, such significant modifications of the transcript sequence and thus its secondary structure may decrease its cellular stability and affect the interaction of protein factors with its polyadenylation signals (mainly β).

The data obtained suggest the following. The polyadenylation efficiency is not decreased when the internal pol III promoter of B2 is replaced by an external one (U6 promoter). Deletion of certain extended regions in the B2 sequence does not decrease the cellular level of B2 transcripts or their polyadenylation efficiency. The acquisition of poly(A) tails by B2 transcripts increases their cellular level up to threefold.

**Figure 3:**
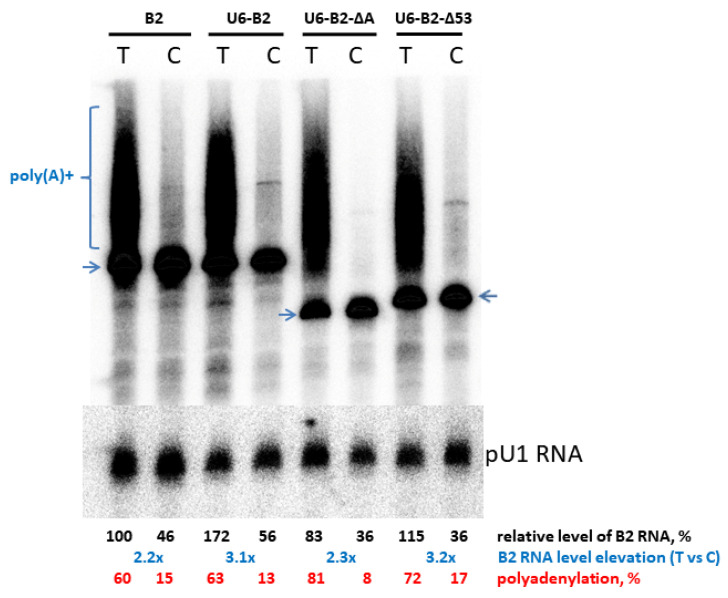
Northern hybridization of RNA from HeLa cells transfected with four types of B2 constructs. Each type included two variants, “T” with the normal (AATAAA) PAS and “C” with the inactivated one (AACAAA). The positions of the primary transcripts are indicated by arrows, while the polyadenylated transcripts are marked by brackets. Normalization was performed using pU1 RNA. The level of B2 RNA relative to the B2_T RNA level is provided below as a percentage. The positive exceedance rates for T over C transcripts are highlighted in blue. The polyadenylation efficiency is shown in red at the bottom.

The latter conclusion was experimentally confirmed by comparing the levels of B2 transcripts with the native (AATAAA) and inactivated (AACAAA) PASs (Figure 3). In all four construct pairs tested, polyadenylation significantly increased the transcript level: from 2.2- (B2) to 3.2-fold (U6-B2-Δ53).

Two other constructs were tested to evaluate the effect of small insertions into specific B2 sites on the polyadenylation of their transcripts. The promoter box B and β-signal are five nucleotides apart, and a 25-bp sequence was inserted into this spacer. This insertion was shown to decrease the polyadenylation efficiency of the transcript 1.5-fold (Figure S2). This confirms the importance of this region for polyadenylation and makes it unsuitable for insertion into a putative B2-based vector. In the second construct, the B2 terminator was inactivated by truncation to TCTT, and a 27-bp sequence ending with a T_6_ transcription terminator was added downstream of it. The polyadenylation efficiency of such a construct decreased 2.6-fold compared to the original construct B2_T (Figure S2). Thus, increasing the distance between the PAS and the 3′ end of the transcript by ~25 nt significantly decreased the polyadenylation efficiency.

### 2.2. Effect of Polyadenylation on Transcript Level of B2 Constructs with a 55-bp Insertion

**Figure 4:**
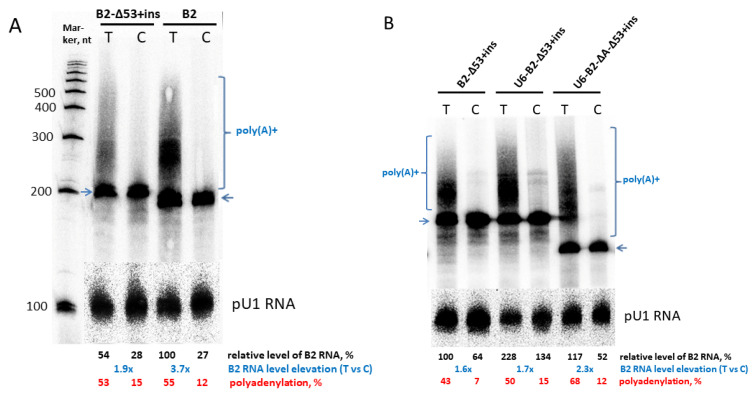
Northern hybridization of RNA from HeLa cells transfected with B2 constructs carrying the 55-bp insertion (ins). The positions of the primary transcripts are indicated by arrows, while the polyadenylated transcripts are marked by brackets. The positive exceedance rates for T over C transcripts are highlighted in blue. The polyadenylation efficiency is shown in red at the bottom. (**A**) Construct with the mouse 5′-flanking sequence. (**B**) Two constructs with the U6 promoter in the 5′-flanking sequence.

The effect of a long foreign sequence insertion into B2 constructs on their polyadenylation efficiency as well as the effect of long poly(A) tails in such transcripts on their cellular level were experimentally tested. A random 55-nt DNA sequence was cloned into the *Eco*RI and *Bam*HI sites of B2T-Δ53 and B2C-Δ53. The resulting constructs B2T-Δ53+ins and B2C-Δ53+ins (Figure 1) were used to transfect HeLa cells and the relative levels of the corresponding transcripts were evaluated as described above (Figure 4A). Polyadenylation was shown to have increased the level of B2-Δ53+ins transcripts 1.4–1.9-fold. This effect of polyadenylation was slightly lower compared to the original B2 construct: the cellular level of polyadenylation-competent transcripts (B2_T) was 2.4–3.7 times higher compared to that of polyadenylation-incompetent transcripts (B2_C).

The same sequence was cloned into the *Eco*RI and *Bam*HI sites of U6-B2-Δ53 and U6-B2-ΔA-Δ53 with the functional PASs (AATAAA) or these sequences inactivated by T to C substitutions (Figure 1). Transfection of HeLa cells with U6-B2-Δ53+ins and U6-B2-ΔA-Δ53+ins showed a 1.7- and 2.3-fold increase in their transcripts levels, respectively (Figure 4B).

**Figure 5:**
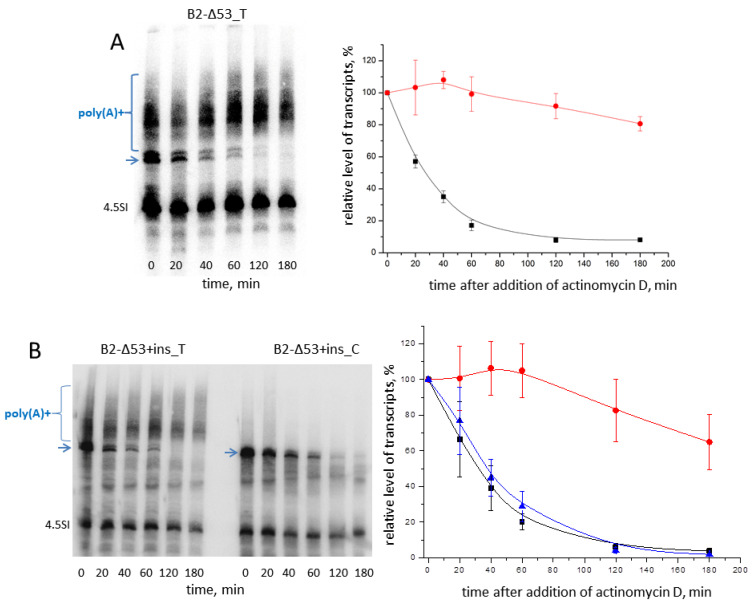
Decay kinetics of B2-Δ53_T transcripts (**A**) as well as B2-Δ53+ins_T and B2-Δ53+ins_C transcripts (**B**). The left and right panels demonstrate Northern hybridizations of cellular RNA and plots of B2 transcript reduction in transfected HeLa cells exposed to the transcription inhibitor, respectively. Cells were also transfected with the mouse 4.5SI RNA gene, whose transcripts are notably stable [35]; 4.5SI RNA was detected by hybridization too. The primary transcripts of B2 constructs are marked with arrows, and the polyadenylated transcripts are in curly brackets. The red line corresponds to the polyadenylated transcripts, while the black line represents the primary (non-polyadenylated) transcript (B2-Δ53_T or B2-Δ53+ins_T). The blue line denotes the polyadenylation incompetent B2-Δ53+ins_C transcript. (Error bars, SD, *n* = 3).

Thus, the results presented in Figure 4 indicate that B2 transcripts with a long insertion can be efficiently polyadenylated (usually at least 50% of the transcripts are polyadenylated) and this significantly (approximately twofold) increases their cellular level. Poly (A) tails protect artificial transcripts from rapid degradation, although the long foreign insertion may decrease their stability. Therefore, we compared the stability of transcripts with and without the 55-nt insertion. HeLa cells were transfected with B2-Δ53_T, B2-Δ53+ins_T, or B2-Δ53+ins_C; 20 h later, cells were exposed to actinomycin D (a transcription inhibitor), and cellular RNA was isolated after different time periods (20, 40, 60, 120 and 180 min). Northern hybridization demonstrated the stability of the polyadenylated B2_T-Δ53 within 180 min of observation (Figure 5A). The polyadenylated transcript with the insertion (B2_T-Δ53+ins) was less stable, its mean level decreased to 65% (Figure 5B). However, even in this case, a significant delay of RNA degradation by the poly(A) tail is observed, since the polyadenylation-incompetent B2_C-Δ53+ins transcript with the half-life of 40 min is completely degraded in 180 min (Figure 5B).

### 2.3. Effect of the U6 Promoter as Well as Deletions and Substitutions on the Level of Ves Transcripts and Their Polyadenylation

A series of constructs based on the Ves SINE from the bat *Myotis daubentonii* were generated (Figure S3A), similar to the B2-based constructs described in Section 2.1. These constructs were used to transfect HeLa cells and the isolated cellular RNA was analyzed by Northern hybridization (Figure S3B). As could be expected, the replacement of the entire box B significantly (eightfold) decreased Ves transcription. Surprisingly, the introduction of the U6 promoter upstream of the Ves SINE with the affected pol III promoter did not restore its transcription. The levels of U6-Ves-subB, U6-Ves-ΔA, and U6-Ves-ΔA-subB transcripts were 16, 12, and 6% of the intact Ves transcript, respectively (Figure S3B). Similarly, a U6 promoter upstream of the native Ves did not increase its transcription (as with B2; Figure 2) but rather decreased it threefold (U6-Ves in Figure S3B). There seems to be an incompatibility between the U6 promoter and the Ves sequences, which prevents the simple development of cassettes combining Ves and the U6 promoter.

On the other hand, this experiment demonstrated efficient polyadenylation of transcripts from constructs with long head deletions (U6-Ves-ΔA and U6-Ves-ΔA-subB) as the initial Ves: 80–84% of the full-length transcripts acquired poly(A) tails (Figure S3B). A substitution of box B reduced the proportion of polyadenylated transcripts (U6-Ves-subB and Ves-subB), but it remained substantial (52 and 60%, respectively). Thus, the data obtained suggest that the structure and length of the head region have a minor effect on the function of the β- and τ-signals in Ves transcripts.

### 2.4. Effect of Polyadenylation on the Level of Ves Transcripts with the 55-bp Insertion

**Figure 6:**
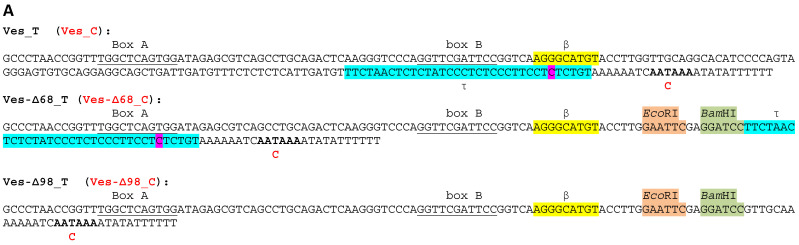

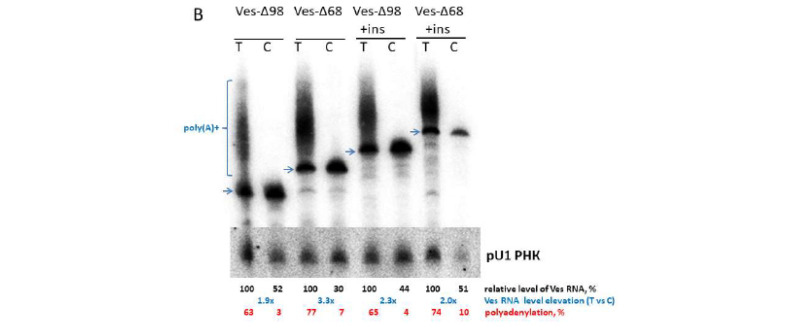
Testing Ves constructs with deletions and a foreign insertion. (**A**) Nucleotide sequences of Ves constructs. Ves_Δ68 and Ves_Δ98 were created by replacing the 68- and 98-bp fragments with regions containing the *Eco*RI (brown) and *Bam*HI (green) sites. The T to C substitution highlighted in purple prevented transcription termination at this site (see Figure S3) and increased the yield of full-length RNA. Boxes A and B of the pol III promoter are underlined. The β- and τ-signals are marked in yellow and blue, respectively. The PAS is indicated in bold. (**B**) Northern hybridization of RNA from HeLa cells transfected with four types of Ves constructs. Each type included two variants, “T” with the normal (AATAAA) PAS and “C” with the inactivated one (AACAAA). Constructs “+ins” contain the foreign 55-bp insertion, the same one as shown in Figure 1. The positions of the primary transcripts are indicated by arrows, while the polyadenylated transcripts are marked by brackets. Normalization was performed using pU1 RNA. The levels of T-constructs were taken as 100% for each construct. The level of B2 RNA relative to the B2_T RNA level is provided below as a percentage. The positive exceedance rates for T over C transcripts are highlighted in blue. The polyadenylation efficiency is shown in red at the bottom.

In the following series of experiments, the possible application of Ves SINE-based constructs for the expression of transcripts with long insertion in transfected cells was tested. First, two constructs were generated. In one construct, the 98-bp spacer between the β-signal and the 3′-terminal A-rich region of Ves was replaced with a 14-bp sequence containing *Eco*RI and *Bam*HI sites (Ves-Δ98, Figure 6A). In the second construct, the 68-bp spacer between the β- and τ-signals was deleted and replaced by a 14-bp sequence containing *Eco*RI and *Bam*HI sites (Ves-Δ68, Figure 6A). Each of these constructs was used to generate variants with PAS inactivated by T to C substitution. The 55-bp fragment (same as in B2 experiments above) was then cloned into *the Eco*RI and *Bam*HI sites of these constructs. All eight constructs were used to transfect HeLa cells and the cellular RNA was analyzed by Northern hybridization. Transcripts of four constructs with the native PAS were efficiently polyadenylated: 63–77% of their molecules had poly(A) tails (Figure 6B). Polyadenylation increased the cellular level of Ves RNA 2.0–3.3-fold, which was also true for the transcripts carrying the foreign 55-bp insertion (Figure 6B); this can be attributed to the increased stability of transcripts with poly(A) tails.

**Figure 7:**
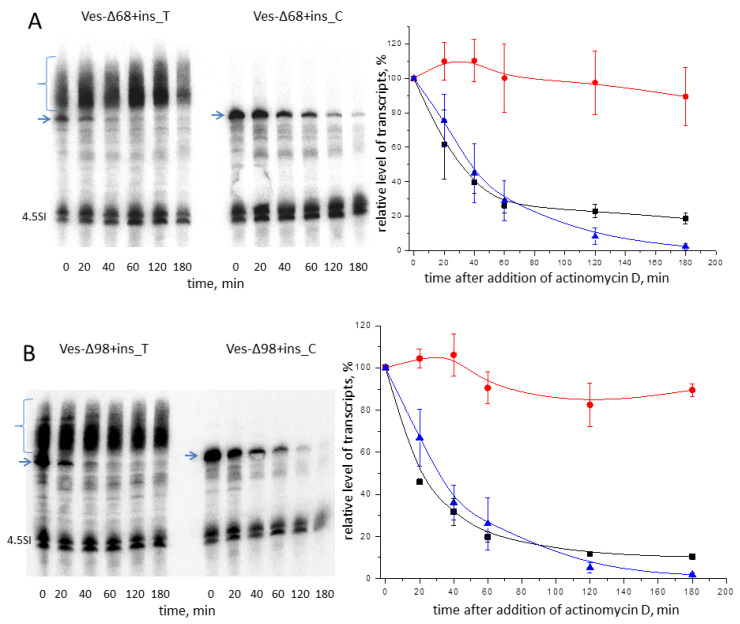
Degradation kinetics of Ves construct transcripts in transfected HeLa cells. (**A**) Ves-Δ68+ins_T and Ves-Δ68+ins_C constructs. (**B**) Ves-Δ98+ins_T and Ves-Δ98+ins_C constructs. The left and right panels demonstrate the Northern hybridization of cellular RNA and plots of Ves transcript reduction in transfected HeLa cells exposed to the transcription inhibitor, respectively. The primary transcripts are indicated by arrows; and polyadenylated transcripts, by curly brackets. The 4.5SI RNA gene was used to co-transfect cells to normalize the level of Ves transcripts. The red line corresponds to the polyadenylated transcripts; the black line, to the primary (non-polyadenylated) transcript (Ves-Δ68+ins_T or Ves-Δ98+ins_T); and the blue line, to the polyadenylation incompetent Ves-Δ68+ins_C and Ves-Δ98+ins_C transcripts. (Error bars, SD, *n* = 3).

The stability of polyadenylated transcripts of Ves-Δ68+ins and Ves-Δ98+ins was tested in experiments similar to those described above for B2-containing constructs. HeLa cells were transfected with one of four plasmids (Ves-Δ68+ins_T, Ves-Δ68+ins_C, Ves-Δ98+ins_T, or Ves-Δ98+ins_C), actinomycin D was added one day later, and cellular RNA was isolated after different periods of time (20, 40, 60, 120, and 180 min). Figure 7 shows the Northern hybridization results for isolated RNA samples and quantitative analysis of the decay kinetics of Ves transcripts. The polyadenylation-incompetent transcripts Ves-Δ68+ins_C and Ves-Δ98+ins_C degraded rapidly (T_0.5_ = 35–40 min), whereas the polyadenylated Ves-Δ68+ins_T and Ves-Δ98+ins_T remained stable within 180 min. Thus, poly(A) tails prolong the life of Ves constructs carrying the long foreign insertion. This results in a higher cellular level of polyadenylated Ves transcripts.

## 3. Discussion

Here we demonstrated the autonomous function of β- and τ-signals in B2 and Ves, i.e., their dependence on other SINE sequences is low, except for the PAS and the transcription terminator, which are strictly required for the polyadenylation of their transcripts. Deletions of the spacer between the β- and τ-signals could even increase polyadenylation efficiency. Deletion of box A together with the spacer between boxes A and B also had no negative effect on the polyadenylation of transcripts. Transcription of SINEs lacking part of the internal pol III promoter could be driven by an external pol III promoter from the 5′-terminal sequence of the U6 RNA gene. Thus, the promoter type does not affect the polyadenylation capacity of the synthesized RNA. Similarly, the substitution of the entire box B did not affect the polyadenylation efficiency of B2; however, such a substitution decreased the polyadenylation of Ves 1.4–1.6-fold. A similar pattern was observed previously [18] for 4-nt substitutions in box B that were not significant for its promoter function. The nucleotide sequence of box B is involved in the function of neighboring β-signal in Ves but not B2. It is plausible that this occurs at the level of RNA folding and exposing of β-signal to protein factors.

According to our experiments, the external U6 promoter could restore the initial transcription level of B2 with deleted box A or substituted box B. A combination of a U6 promoter with an intact internal promoter increased B2 transcription 1.7–2.3-fold. It is conceivable that independent transcription from both pol III promoters combined to double the transcript level. Surprisingly, a similar construct with Ves containing a U6 promoter showed a threefold decrease in transcription relative to Ves with the original genomic 5′-terminal sequence. It can only be speculated that U6 promoter-binding protein factors interfere with those that bind the internal promoter of Ves. Similarly, the combination of pol III promoter boxes (A, B, and TATA-like) in the genes of two small nuclear RNAs significantly reduced the activity of such chimeric promoters [34]. This could be attributed to an inconsistency between the boxes of one gene and the nucleotide sequence of another gene. Furthermore, the U6 promoter initiated approximately one-tenth as many Ves transcripts lacking boxes A and B as the original Ves SINE. The modified Ves sequence downstream of the U6 promoter could have attenuated its function. Alternatively, these Ves sequence modifications could reduce transcript stability in the cell. As previously noted, RNA synthesis from the U6 promoter significantly increased for transcripts that began with the 27-nt sequence specific to the 5′ end of U6 RNA [28]. The authors assumed that this 27-nucleotide sequence with a hairpin structure stabilizes RNA increasing its cellular level. Probably the cellular level of Ves RNA transcribed from the U6 promoter can be significantly increased by inserting this sequence at its 5′ end. We have currently excluded expression cassette development that involves Ves constructs with the U6 promoter.

Our primary concept behind developing T^+^ SINE-based expression cassettes was that their transcripts can be polyadenylated leading to an increase in their longevity and cellular level. The experimental data presented demonstrate that B2-based constructs showed a 2.2–3.2-fold increase in expression level compared to similar constructs with mutant PASs (Figure 3 and Figure 4). Similar results were obtained in the experiments involving Ves constructs, where the transcripts with polyadenylation competence showed 1.9–3.3 times greater expression levels compared to those without it (Figure 6). In the case of B2 and Ves constructs with the random 55-bp insertion, the level of polyadenylated transcripts increased 1.6–2.3-fold compared to non-polyadenylated transcripts (Figure 4 and Figure 6). The stability of polyadenylated transcripts of Ves constructs with and without the 55-bp insertion was similar (Figure 7). The insertion made the polyadenylated B2 transcripts marginally less stable than those without it (Figure 5), which can explain the moderate effect of polyadenylation on the level of such transcripts, namely 1.6–1.9 times (Figure 4). The implemented insertion could exacerbate the susceptibility of B2 transcripts to degradation. Shorter insertions may not negatively impact the transcript stability of B2 constructs. The data obtained for Ves constructs showed more promising results in their application as cassettes expressing short insertions. Pol III actively transcribes such cassettes and synthesizes RNA which becomes stable in the cell through polyadenylation. Furthermore, poly(A) tails can enable their principal localization in the cytoplasm. This localization can enhance the impact of RNA expression on the cell. For example, this effect can be anticipated in cassettes that express a microRNA sponge that reduces its cellular level [36].

## 4. Materials and Methods

### 4.1. Plasmid Constructs

SINE-containing constructs B2_T, B2_C, Ves_T, and Ves_C were obtained and described previously [18,22]. Constructs with the 5′-flanking sequence of SINE replaced with a U6 promoter were generated using a two-step PCR. The first step included the amplification of (1) a 262-bp fragment containing the pol III promoter of the human U6 RNA (from vector psiSTRIKE hMGFP) and (2) a SINE without the 5′-flanking sequence (from B2_T or Ves_T). The reverse primers used in the former PCR and direct primers used in the latter had overlapping nucleotide sequences. In the second step, the two amplification products were mixed to be used in PCR-generating products with the U6 promoter and a SINE (B2 or Ves), which were cloned into pGEM-T (Promega, Madison, WI, USA). Deletions, short insertions, and nucleotide substitutions were introduced into B2- or Ves-containing plasmids using the Phusion Site-Directed Mutagenesis Kit (Thermo Fisher Scientific, Waltham, MA, USA). The 55-bp sequence was produced using a random sequence generator and ligated into *Eco*RI and *Bam*HI sites of B2-Δ53, Ves-Δ68, or Ves-Δ98 plasmids.

The control plasmid pU1 used to normalize transcripts of SINE constructs in transfection experiments was described elsewhere [34]. The plasmids designed for transfection were isolated using the Plasmid Midi Kit (Qiagen, Hilden, Germany) according to the manufacturer’s protocol.

### 4.2. Cell Transfection and Northern Blot Analysis

HeLa cells (ATCC, CCL-2) were grown to an 80%-confluent monolayer in 60 mm Petri dishes using DMEM with 10% fetal bovine serum. Cells on each dish were co-transfected with 4 μg DNA of B2 or Ves constructs and 1 μg of the control pU1 mixed with 10 μL of TurboFect reagent (Thermo Fisher Scientific, Waltham, MA, USA) according to the manufacturer’s protocol. (In experiments on the stability of transcripts of B2 and Ves constructs, pU1 was replaced with 1 μg of plasmid with the mouse 4.5SI RNA gene [37], and the transcription inhibitor actinomycin D (5 μg/mL) was added to the medium 20 h after transfection. See details in [23]). Transfections were performed in three replicates. The cellular RNA was isolated 20 h after transfection (or after additional treatment of cells by actinomycin D for 20, 40, 60, 120, or 180 min) using the guanidine-thiocyanate method [38] and further purified by RNase-free DNase I treatment [22]. RNA samples (10 μg) obtained from each transfection were separated by electrophoresis in 6% polyacrylamide gel with 7 M urea, transferred to a nylon membrane (GVS, Bologna, Italy) using semi-dry electroblotting, and hybridized with probes labeled using α[^32^P]-dATP, Taq-polymerase, and reverse primers [18]. Figure S4 shows primer positions in the probe nucleotide sequences. The probes corresponding to the 5′-terminal portions of insertions were used for hybridization detection of SINE transcripts with long deletions in their 3′-terminal parts; and vice versa, the 3′-terminal portions were labeled to detect SINEs with 5′-terminal deletions. If a membrane contained SINE transcripts with deletions in 5′- and 3′-terminal parts, both labeled probes were mixed in equal proportions (see Figure 2 and Figure 3). The Northern hybridization conditions were described elsewhere [18]. Hybridization signals were quantified by scanning the membranes in a Phosphorimager (Image Analyzer Typhoon FLA 9000; GE Healthcare Bio-sciences, Uppsala, Sweden). OptiQuant 3.0 program was used for the quantitative analysis of images. Radioactive signal was measured for the full-length primary SINE transcript (the band of the longest RNA) and for the polyadenylated transcript (an even longer RNA looking like a smear). Finally, the percentage of polyadenylation was calculated for the transcripts of each SINE construct.

## Data Availability

Not applicable.

## Supplementary Materials

The following supporting information can be downloaded at: https://www.mdpi.com/article/10.3390/ijms241914600/s1.
