# Supplementary material for: SINEs as Potential Expression Cassettes: Impact of Deletions and Insertions on Polyadenylation and Lifetime of B2 and Ves SINE Transcripts Generated by RNA Polymerase III
Source: Int J Mol Sci. 2023 Sep 27;24(19):14600. doi: 10.3390/ijms241914600 (PMC10572872; doi:10.3390/ijms241914600)
Supplement: Supplementary file 1 [file ijms-24-14600-s001.zip › ijms-2607224-supplementary.pdf]

5’-Flanking sequence of human U6 RNA gene (U6 promotor):

tcg
-260

ggcaggaagagggcctatttcccatgattccttcat**atttgcat**atacgatacaaggctgttagagagataattagaattaatttg
-250 -240 -230 -220 -210 -200 -190 -180

actgtaaacacaaagatattagtacaaaatacgtgacgtagaaagtaataatttcttgggtagtttgcagttttaaaattatgttt
-170 -160 -150 -140 -130 -120 -110 -100 -90

taaaatggactatcatatg**cttaccgtaacttgaaagta**tttcgatttcttggc**tttatatat**cttgtggaaaggacgaaacaccg
-80 -70 -60 -50 -40 -30 -20 -10 -1

Mouse 5’-flanking sequence of SINE B2 copy studied:

actcaggggcagtgtacacacttagcttgaccaatcttgagtatagtttatatt**aaaatagtg**acttcaagaaaatattaagtcgg
-80 -70 -60 -50 -40 -30 -20 -10 -1

Bat (*Myotis daubentonii*) 5’-flanking sequence of SINE Ves copy studied:

t**tcttaagt**tatcagtaaaaatgtcacataag
-30 -20 -10 -1

Figure S1. 5’-Flanking sequences of the murine B2 (*Mus musculus*) and bat Ves (*Myotis daubentonii*) genomic copies as well as the human small nuclear U6 RNA gene (GenBank accession number: X07425.1) used in this work. The latter includes a pol III promoter [21, 23], which was used to replace the native 5’-flanking sequences of B2 and Ves constructs [13] with names starting with “U6” (see Figures 1 and S3). Functional regions of the U6 promoter are colored: TATA box, green; proximal sequence element, red; and distal sequence element, blue. Potential TATA-like boxes (positions –31 to –24) in the 5'-terminal sequences of B2 and Ves are shown in purple.

A

B2_T; (B2_C)

```
                  box A                                               box B             β
GGGCTGGAGAGATGGCTCAGCGGTTAAGAGCACTGACTGCTCTTCCGAAGGTCCTGAGTTCAATTCCCGGCAACCACATGGTGGCT
CACAACCATCCGTAATGAGATCTGGTGCCCTCTTCTGGAGTGTCTGAGGACAGCTACAGTGTACTTACATATAATAAATAAATCAA
CAAATCTTTTT                                                          τ           C   C
```

B2+ins25nt@B/β

```
                  box A                                               box B        EcoRI      BamHI
GGGCTGGAGAGATGGCTCAGCGGTTAAGAGCACTGACTGCTCTTCCGAAGGTCCTGAGTTCAATTCCCAAGAATTCGTCAGGATCC
GGTCACGGGCAACCACATGGTGGCTCACAACCATCCGTAATGAGATCTGGTGCCCTCTTCTGGAGTGTCTGAGGACAGCTACAGTG
TACTTACATATAATAAATAAATCAACAAATCTTTTT                                                 τ
```

B2+ins27nt@end

```
                  box A                                              box B              β
GGGCTGGAGAGATGGCTCAGCGGTTAAGAGCACTGACTGCTCTTCCGAAGGTCCTGAGTTCAATTCCCGGCAACCACATGGTGGCT
CACAACCATCCGTAATGAGATCTGGTGCCCTCTTCTGGAGTGTCTGAGGACAGCTACAGTGTACTTACATATAATAAATAAATCAA
CAAATCTTAAAGGATCCGTCAGAATTCAATTTTTTT                                 τ
           BamHI     EcoRI
```

B

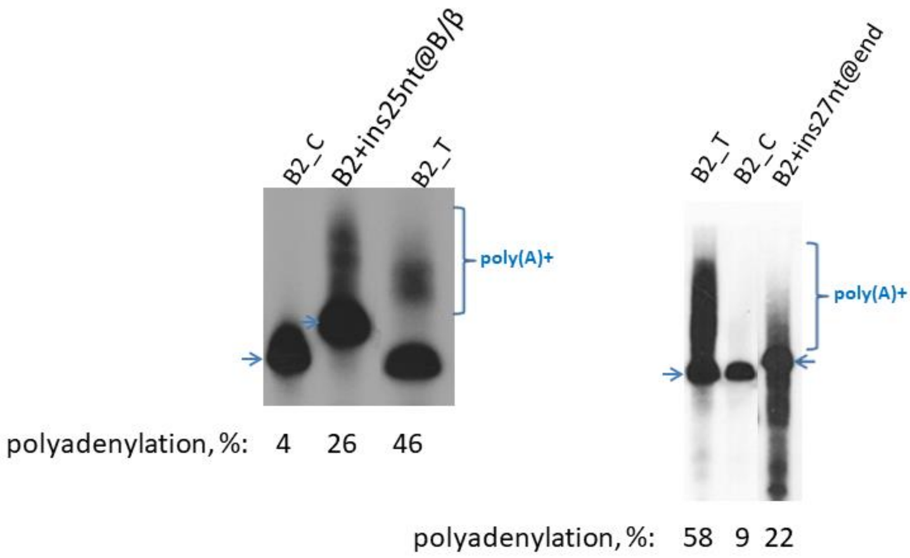

Figure S2. Testing B2 constructs with a short insertion between box B and β-signal (B2+ins25nt@B/β) or at the end of SINE (B2+ins27nt@end). (**A**) Nucleotide sequences of B2_T, B2_C, B2+ins25nt@B/β, and B2+ins27nt@end constructs. The 25-nt and 27-nt insertions are shown in blue; they contain *Eco*RI and *Bam*HI sites. A truncated (inactivated) transcription terminator in the B2+ins27nt@end construct is double-underlined. (**B**) Results of the Northern hybridization of RNA from HeLa cells transfected with these constructs.

A

Ves_T (Ves_C):

Box A / box B / β

GCCCTAACCGGTTTGGCTCAGTGGATAGAGCGTCAGCCTGCAGACTCAAGGGTCCCAGGTTCGATTCCGGTCAAGGGCATGTACCTTGGTTGCAGGC
ACATCCCCAGTAGGGAGTGTGCAGGAGGCAGCTGATTGATGTTTCTCTCTCATTGATGTTTCTAACTCTCTATCCCTCTCCCTTCCTTTCTGTAAAA
AATC**AATAAA**ATATATTTTTT

τ

C

Ves-subB_T, U6-Ves-subB_T (U6-Ves-subB_C):

Box A / sub-box B / β

GCCCTAACCGGTTTGGCTCAGTGGATAGAGCGTCAGCCTGCAGACTCAAGGGTCCCATCCGTCCTGAGGGTCAAGGGCATGTACCTTGGTTGCAGGC
ACATCCCCAGTAGGGAGTGTGCAGGAGGCAGCTGATTGATGTTTCTCTCTCATTGATGTTTCTAACTCTCTATCCCTCTCCCTTCCTTTCTGTAAAA
AATC**AATAAA**ATATATTTTTT

τ

C

U6-Ves-ΔA_T (U6-Ves-ΔA_C):

Δ-box A / box B / β

GCCCTAACCGGTT--GGTTCGATTCCGGTCAAGGGCATGTACCTTGGTTGCAGGC
ACATCCCCAGTAGGGAGTGTGCAGGAGGCAGCTGATTGATGTTTCTCTCTCATTGATGTTTCTAACTCTCTATCCCTCTCCCTTCCTTTCTGTAAAA
AATC**AATAAA**ATATATTTTTT

τ

C

U6-Ves-ΔA-subB_T (U6-Ves-ΔA-subB_C):

Δ-box A / sub-box B / β

GCCCTAACCGGTT--TCCGTCCTGAGGGTCAAGGGCATGTACCTTGGTTGCAGGC
ACATCCCCAGTAGGGAGTGTGCAGGAGGCAGCTGATTGATGTTTCTCTCTCATTGATGTTTCTAACTCTCTATCCCTCTCCCTTCCTTTCTGTAAAA
AATC**AATAAA**ATATATTTTTT

τ

C

Figure S3A. Nucleotide sequences of constructs with Ves SINE. Boxes A and B of pol III promoter are underlined; β- and τ-signals are marked in yellow and blue, respectively; substituted nucleotides are shown in red; dashes indicate deleted nucleotides. In the constructs whose names start from “U6,” the 5’-flanking sequence of the particular Ves copy was replaced with the pol III promoter of the human U6 RNA gene (their nucleotide sequences are given in Figure S1). PAS is boldfaced. The names of constructs with PAS inactivated by T to C substitutions end with “C” and are shown in red. The site within the polypyrimidine motif where transcription often terminates (such transcript is marked by an asterisk in Fig. S3B) is wavy-underlined.

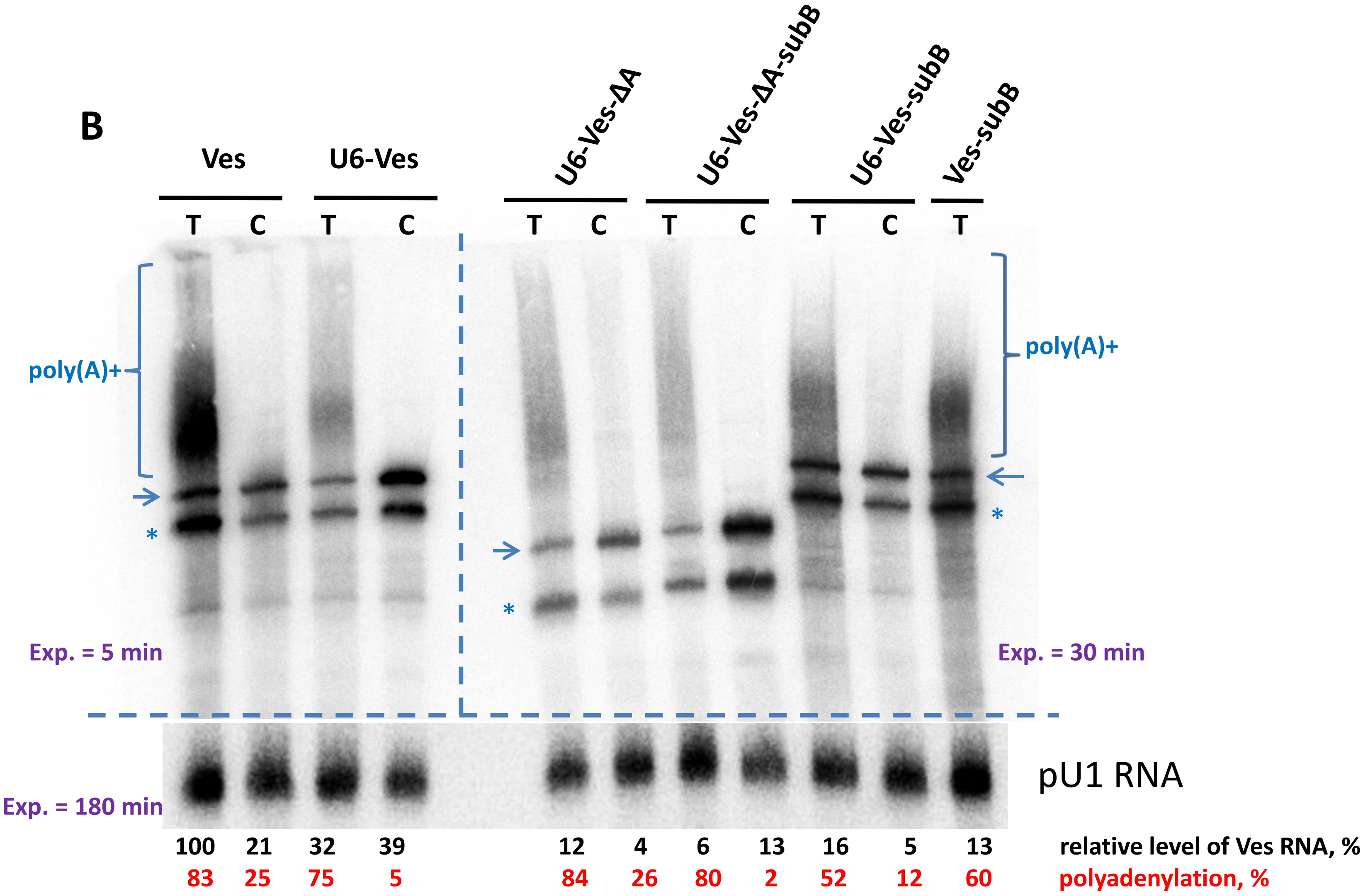

Figure S3B. Northern hybridization of RNA from HeLa cells transfected with Ves constructs shown in Figure S3A. RNAs of constructs with the conventional (AATAAA) or inactivated (AACAAA) PASs are marked by letters T and C above the lanes, respectively. The arrows and asterisks mark the positions of the primary full-length and non-full-length transcripts, respectively; and curly brackets mark the polyadenylated transcripts. PU1 RNA was used for normalization. Different parts of the hybridization membrane were exposed for different time periods (shown in purple). The relative level of Ves RNA is shown below (Ves_T was taken as 100%). Polyadenylation efficiencies are shown in red (transcripts marked by asterisks were excluded).

A

Full-length B2 probe

GGGGCTGGAGAGA**TGGCTCAGCGG**TTAAGAGCACTGACTGCTCTTCCGAAGGTCCTG**AGTTCAATTCCC**CG

GCAACCACATGGTGGCTCACAACCATCCGTAATGAGATCTGGTGCCCTCTTCTGGAGTGTCTGAGGACAG

CTACAGTGTACTTACATATAATAAATAAATCAACAAATCTTTT

B

5'-Half B2 probe

GGGGCTGGAGAGA**TGGCTCAGCGG**TTAAGAGCACTGACTGCTCTTCCGAAGGTCCTG**AGTTCAATTCCC**CG

GCAACCACATGGTGGCTCACAACCATCCGTAATGAGATCTGGTGCCCTCTTCTGGAGTGTCTGAGGACAG

CTACAGTGTACTTACATATAATAAATAAATCAACAAATCTTTT

C

Full-length Ves probe

GCCCTAACCGGTT**TGGCTCAGTGG**ATAGAGCGTCAGCCTGCAGACTCAAGGGTCCCA**GGTTCGATTCC**GG

TCAAGGGCATGTACCTTGGTTGCAGGCACATCCCCAGTAGGGAGTGTGCAGGAGGCAGCTGATTGATGTT

TCTCTCTCATTGATGTTTCTAACTCTCTATCCCTCTCCCTTCCTTTCTGTAAAAAATCAATAAAATATAT

TTTTT

D

5'-Half Ves probe

GCCCTAACCGGTT**TGGCTCAGTGG**ATAGAGCGTCAGCCTGCAGACTCAAGGGTCCCA**GGTTCGATTCC**GG

TCAAGGGCATGTACCTTGGTTGCAGGCACATCCCCAGTAGGGAGTGTGCAGGAGGCAGCTGATTGATGTT

TCTCTCTCATTGATGTTTCTAACTCTCTATCCCTCTCCCTTCCTTTCTGTAAAAAATCAATAAAATATAT

TTTTT

E

"Ins" probe

AATTCAACTATCCTGTGAACGTTGGATGGATGGAATGACATGCCTCTAGGGCACGCTGTAG

Figure S4. Probes used to detect B2 and Ves constructs by Northern hybridization. Boxes A and B are boldfaced. Full-length (**A**, **C**) and 5'-half (**B**, **D**) probes are shown in green and yellow, respectively. (**E**) The "ins" probe corresponding to the 55-bp insertion is marked blue. The blue and red arrows indicate the positions of the direct and reverse primers, respectively, used for probe amplification. Probe labeling was performed by 25 PCR cycles with the reaction mixture (25 µl) containing ~2 ng of DNA probe, 10 pmole reverse primer, 5 U Taq polymerase, 1 MBq $\alpha[^{32}P]$-dATP, and 0.5 mM dGTP, dCTP, and TTP each. Since dATP concentration was very low, the synthesized labeled DNA strands were relatively short and largely included only 3'-terminal halves of SINEs, when full-length probes were templates.

The probes were used in Northern hybridizations presented in the following figures.
Figures 2 and 3: full-length B2 probe mixed with 5’-half B2 probe.
Figures 4A and 5: 5’-half B2 probe.
Figure 4B: ins probe.
Figures 6 and 7: 5’-half Ves probe.
Figure S3B: full-length Ves probe.
